# Tuberculosis (TB) treatment challenges in TB-diabetes comorbid patients: a systematic review and meta-analysis

**DOI:** 10.1080/07853890.2024.2313683

**Published:** 2024-02-12

**Authors:** Mahnoor Khattak, Anees ur Rehman, Tuba Muqaddas, Rabia Hussain, Muhammad Fawad Rasool, Zikria Saleem, Mesfer Safar Almalki, Samar Adel Alturkistani, Shuruq Zuhair Firash, Oseid Mohammed Alzahrani, Ammar Abdulraheem Bahauddin, Safa Almarzooky Abuhussain, Muath Fahmi Najjar, Hossameldeen Mahmoud Ali Elsabaa, Abdul Haseeb

**Affiliations:** a Department of Pharmacy Practice, Faculty of Pharmacy, Bahauddin Zakariya University, Multan, Pakistan; b Discipline of Social and Administrative Pharmacy, School of Pharmaceutical Sciences, University Sains Malaysia, Penang, Malaysia; c Security Forces Hospital, Ministry of Interior, Makkah, Saudi Arabia; d Department of Clinical Pharmacy, College of Pharmacy, Umm Al-Qura University, Makkah, Saudi Arabia; e Department of Pharmacology and Toxicology, College of Pharmacy, Taibah University, Madinah, Saudi Arabia; f Department of Clinical Pharmacy, Al Rayan Private College of Health Sciences and Nursing, Madinah, Saudi Arabia

**Keywords:** Diabetes mellitus, tuberculosis, comorbidity, extended treatment duration, recurrence, disease progression

## Abstract

**Background:**

The Directly Observed Treatment-Short Course (DOTS) Programme was implemented by WHO and includes a combination of four anti-tuberculosis (TB) drugs (isoniazid, pyrazinamide, ethambutol and rifampicin) for a period of six months to eradicate the TB infection completely. Diabetes mellitus (DM) is recognized as one of a strong contributor of TB according to World Health Organization (WHO). The presence of diabetes mellitus type 2 (DM type 2) makes TB treatment complicated. Thus, the objective of the current meta-analysis was to identify and quantify the impact of type 2 DM on treatment outcomes of TB patients treated under the DOTS Programme.

**Methods:**

This meta-analysis was performed according to the Preferred Reporting Items for Systematic Reviews and Meta-Analyses (PRISMA) guidelines. Through a systematic review of relevant literature, we focused on studies investigating treatment outcomes including extended treatment duration and recurrence for individuals with both TB and DM undergoing DOTS therapy. The extracted information included study designs, sample sizes, patient characteristics and reported treatment results.

**Results:**

In 44 studies from different parts of the world, the pooled HR for the impact of DM on extended treatment duration and reoccurrence were HR 0.72, 95% CI 0.56–0.83, *p* < .01 and HR 0.93, 95% CI 0.70–1.04, *p* = .08, respectively. The pooled HR for impact of DM on composite TB treatment outcomes was calculated as 0.76 (95% CI 0.60–0.87), *p* < .01 with an effect size of 41.18. The heterogeneity observed among the included studies was moderate (*I*^2^ = 55.79%).

**Conclusions:**

A negative impact of DM was found on recurrence and extended treatment duration in TB patients treated with DOTS therapy. DM type 2 is responsible for the TB treatment prolongation and TB recurrence rates. By implementing effective management strategies and advancing research, the challenges can be mitigated, arising due to the complex interaction between DM and TB.

## Introduction

Tuberculosis (TB) infection is a serious global health problem. According to World Health Organization (WHO), approximately 5.8 million people were diagnosed with TB and almost 1.5 million people experienced death due to TB [1]. Diabetes mellitus (DM) is recognized as one of a strong contributor of TB according to WHO [2]. Diabetes mellitus and TB co-existence has become a major health concern worldwide [3]. The presence of DM may be responsible for increasing the severity of TB disease [4]. Patients with DM are three times more susceptible to TB as compared to the normal population [3]. Diabetes mellitus is becoming more prevalent in various regions of the world [4]. The estimated global prevalence of TB-DM comorbid patients was 13.73% [5]. The WHO highlights that DM worsens treatment outcomes for TB and thus causes TB disease progression [6]. DM is responsible for extended treatment duration, lower treatment success rates [7], high risks of recurrence or relapse, drug resistance [8] and even death in TB patients [7, 9]. DM accounts for approximately 11% of deaths in TB patients worldwide [10]. Controlling TB-DM comorbid conditions can enhance TB treatment success rates by reducing the risk of TB treatment prolongation, death, TB recurrence and drug resistance. It can also reduce the risk of complications caused by DM comorbidity in TB patients, thus improving patient quality of life [11]. Due to the presence of DM in TB patients, TB treatment has become a challenge [12].

The WHO and the International Union Against TB and Lung Disease (IUATLD) framework aims to reduce the dual burden of TB and DM in affected populations through mutual efforts and developing effective treatment approaches [13]. Thus, the Directly Observed Treatment-Short Course (DOTS) Programme was introduced by WHO in 1993 and implemented in 187 countries in 2005 [14]. Approximately, 4.9 million TB patients were treated under the DOTS Programme during the implementation year [14]. It makes sure that patients adhere to their medications and aims to enhance TB treatment success rates [15]. The DOTS strategy includes a combination of four anti-TB drugs (isoniazid, pyrazinamide, ethambutol and rifampicin) for a period of six months to completely eradicate the TB infection [16]. The presence of DM makes TB treatment complicated and is responsible for the extended treatment duration [17]. It is suggested that the duration of TB treatment may extend from six months to nine months due to the presence of DM [17]. Thus, it is necessary that DM be confirmed earlier to prevent TB progression in TB patients [18].

Previous systematic review and meta-analysis reported the impact of DM on TB treatment results. There were following limitations present in these earlier systematic review and meta-analysis: unadjusted covariates [19], small sample size, not specifically focused on type 2 DM [20], and no specified therapy guidelines [21]. No previous review specifically assessed the impact of type 2 DM on TB patients treatment outcomes including extended treatment duration and recurrence following the DOTS Programme for TB treatment. Thus, keeping in mind the limitations of the previous systematic reviews and meta-analysis, the objective of the current meta-analysis was to identify and quantify the impact of type 2 DM on treatment outcomes of TB patients treated under the DOTS Programme.

## Methodology

### Search strategy and study selection

This meta-analysis was performed according to the Preferred Reporting Items for Systematic Reviews and Meta-Analyses (PRISMA) guidelines [22]. The databases PubMed, Google Scholar, EMBASE, Web of Science and Cochrane library were searched (till June 2023) for studies reporting the DM impact on TB treatment outcomes in which the treatment regimen given to TB patients was DOTS therapy recommended by WHO guidelines and the outcomes were defined by WHO criteria. According to PICOs, the following Mesh terms were used to extract relevant articles: ‘Diabetes Mellitus’ [Mesh] OR ‘Diabetes Mellitus, Type 2’ [Mesh] AND ‘Tuberculosis’ [Mesh] OR ‘Tuberculosis, Pulmonary’ [Mesh] AND ‘TB treatment’ OR ‘TB patients without Type 2 Diabetes Mellitus’ OR ‘Treatment Outcome’ [Mesh] AND ‘Risk Factors’ [Mesh] OR ‘extended treatment duration’ OR ‘recurrence’. The references provided at the end of each included study were also searched for inclusion of relevant studies in this meta-analysis. Only English-language articles were considered.

#### Inclusion criteria

The studies were included in this meta-analysis based on the following PICOs criteria: (1) adult patients with diagnosis of TB, involving both TB-diabetes mellitus type 2 (DM type 2) comorbid patients and alone TB patients. (2) Research articles in which the treatment regimen given to TB patients was DOTS therapy recommended by WHO. (3) Research articles comparing DM impact on TB treatment outcomes including extended treatment duration and recurrence in TB-DM comorbid patients vs. TB patients only. (4) Research articles in which patients had their data reported on any of the following TB treatment outcomes, unsuccessful: extended treatment duration and recurrence. (5) Research articles having a prospective and retrospective cohort, cross-sectional or case-control study design. (6) Original research articles are published in English only.

#### Exclusion criteria

The studies were excluded from this meta-analysis: (1) if they were non-human studies, studies involving children, pregnant women and studies involving patients with any critical illness. (2) Studies involving patients using different anti-TB therapies, patients receiving any type of integrated care. (3) Studies analysing type 1 DM patients. (4) Studies analysing sputum culture conversion only. (5) Non-research articles, case reports, case series, models and editorials. (6) Studies for which no full text was available and studies other than English language.

The articles were reviewed on the basis of inclusion and exclusion criteria by two reviewers independently. The third reviewer reviewed the extracted data. Conflicts, if any, were then resolved through discussion with a fourth reviewer, if needed.

### Data extraction and quality assessment

The data that were extracted from the included studies by two reviewers independently in a data extraction form are as follows: author name, country, publication year of the study, study design, study duration, sample size (TB patients, TB-DM patients), covariates and TB treatment outcome assessed. The data extraction form was then reviewed and verified by a third reviewer, and conflicts were discussed with a fourth reviewer and sorted out for consensus until a final decision was taken. The summaries of the included studies are provided in Table 1. The quality of the studies included in this meta-analysis was checked individually by using the Newcastle-Ottawa Scale (NOS) [63]. The NOS examines potential bias in three different domains: selection of study groups (four points), group comparability (two points) and outcome assessment (three points), assigning greater points for a lower likelihood of bias in each of these domains, up to a maximum of nine points. A score of six or greater indicated less bias and high study quality.

**Table 1.** Summaries of the included studies.

Reference	Country	Research design	Study duration	TB patients	TB-DM comorbid patients	Inclusion criteria	Covariates	Unsuccessful treatment outcomes assessed
Adane et al. [2]	Ethiopia	Prospective	2020–2021	267	24	Patients on first-line anti-TB treatment	Age, BMI, gender, smoking, alcohol	Extended treatment duration
Chang et al. [23]	Taiwan	Prospective	2004–2005	192	60	Patients followed treatment recommendations	NR	Extended treatment duration
Eksombatchai et al. [1]	South Korea	Retrospective	2017–2020	199,571	47,952	TB patients who completed TB treatment successfully	Age, region, household income, nationality, TB lesions, previous TB history, AFB smear, disability, CCI scores	Recurrence
Viswanathan et al. [24]	India	Retrospective	NR	245	96	TB-DM comorbid patients for analysis	NR	Extended treatment duration
Satung et al. [25]	Thailand	Retrospective	2010–2012	7805	555	Patients who were smear positive before treatment	Age, sex, occupation, comorbidity, sputum smear, DM	Extended treatment duration
Alisjahbana et al. [26]	Indonesia	Prospective	2000–2005	634	94	TB-DM comorbid patients	NR	Extended treatment duration
Ghanta et al. [27]	India	Prospective	NR	100	50	TB-DM comorbid patients	NR	Extended treatment duration
Jiménez-Corona et al. [28]	Mexico	Prospective	1995–2010	1262	374	TB-DM comorbid patients	Gender, smoking, HIV infection, BMI	Extended treatment duration, recurrence
Yoon et al. [29]	South Korea	Prospective	2012–2014	661	157	TB-DM comorbid patients with age ≥ 18 years	Age, BMI, smoking, DM status, presence of comorbidity, sputum positive smear	Extended treatment duration
Prakash [30]	India	Retrospective	NR	160	80	TB-DM comorbid patients with age ≥ 18 years	NR	Extended treatment duration
Kang et al. [31]	South Korea	Retrospective	2000–2002	1407	239	MDR-TB patients	NR	Extended treatment duration
Siddiqui et al. [32]	India	Prospective	2014	316	50	TB patients with age more than 15 years and receiving DOTS therapy	Age, gender, BMI, TB history, clinical presentation	Extended treatment duration
Barss et al. [33]	Canada	Retrospective	2007–2012	690	136	Patients with age ≥18 years, appropriate clinical charts	Age, ethnicity, immunocompromised state	Extended treatment duration, recurrence
Delgado-Sánchez et al. [34]	Mexico	Retrospective	2000–2012	181,378	34,988	TB patients with age ≥20 years	Age, gender, previous TB treatment, malnutrition	Extended treatment duration
Ayeni et al. [35]	Nigeria	Retrospective	2011–2012	424	36	Patients with age >18 years	NR	Extended treatment duration
Kornfeld et al. [36]	India	Prospective	2014–2018	389	256	Pulmonary TB patients with age 25–60 years	Age, gender, height, smoking, income, alcohol intake	Extended treatment duration
Gil-Santana et al. [37]	Brazil	Retrospective	2004–2010	244	128	TB-DM comorbid patients and TB-non-DM patients with age ≥ 18 years	Age, gender	Extended treatment duration
Magee et al. [38]	Georgia	Retrospective	2009–2011	1349	72	Patients with age ≥18 years, patients with confirmed MDR-TB	Age, gender, BMI, smoking, alcohol, HIV, previous TB treatment, cavitary disease, disseminated TB	Extended treatment duration
Mukhtar and Butt [39]	Pakistan	Prospective	NR	614	113	Patients with age ≥15 years, no prior intake of ATT	Age, smoking, BMI, area of residence	Extended treatment duration
Chiang et al. [40]	Taiwan	Retrospective	2005–2010	1473	705	Culture positivity in TB patients, patients with DM history	Age, gender, sputum smear, drug resistance, smoking	Extended treatment duration
Magee et al. [41]	Georgia	Prospective	2011–2014	318	37	Patients with age 7–35 years, new TB cases, HbA1c tested, eligible for standard treatment	Age, gender, HIV infection, smoking	Extended treatment duration
Muñoz-Torrico et al. [42]	Mexico	Retrospective	2010–2015	90	49	MDR-TB and XDR-TB patients	NR	Extended treatment duration
Perez-Navarro et al. [43]	Mexico	Prospective	2006–2014	507	183	Patients with MDR-TB, prior DM diagnosis	Age, gender, overcrowding, smoking	Extended treatment duration, recurrence
Sembiah et al. [44]	India	Prospective	2014–2017	662	82	Adult patients with age ≥18 years	NR	Extended treatment duration
Arriaga et al. [45]	Brazil	Prospective	2015–2019	643	107	Patients with pulmonary TB, age ≥18 years, treatment completion	Age, gender, alcohol, HIV infection, smoking	Extended treatment duration
Sulaiman et al. [46]	Malaysia	Retrospective	2006–2007	1267	338	Registered TB patients	NR	Extended treatment duration, Recurrence
Rout et al. [47]	India	Case control	2019–2020	120	60	Patients with age 18–64, received treatment	NR	Extended treatment duration
Leung et al. [17]	China	Prospective	2006–2010	21,414	3331	Patients treated at clinics	Age, gender, ethnicity, residence, employment, alcohol, smoking, HIV, previous TB treatment	Recurrence
Sahakyan et al. [48]	Armenia	Retrospective	2013–2014	621	36	adult TB patients	Weight, sputum smear	Extended treatment duration
Lee et al. [49]	South Korea	Retrospective	2010–2012	1044	252	Patients with age >30 years, diagnosed pulmonary TB	NR	Extended treatment duration, recurrence
Haile Workneh et al. [50]	Ethiopia	Prospective	2013–2015	1314	109	TB patients with age ≥15 years, completed TB treatment	Age, gender, BMI, HIV infection area of residence, adherence to TB treatment	Extended treatment duration
You et al. [51]	China	Retrospective	2017	89,788	335	TB-DM patients, age ≥18 years	NR	Extended treatment duration
Hongguang et al. [52]	China	Prospective	2010–2011	1126	182	Patients with confirmed PTB diagnosis	NR	Extended treatment duration, recurrence
Wang et al. [7]	Taiwan	Retrospective	2003–2006	217	74	Patients with diagnosed and confirmed pulmonary TB	Age, gender	Extended treatment duration
Mahato et al. [53]	Nepal	Prospective	NR	408	102	Patients with diagnosed TB, undergoing TB treatment	Age, employment, history of TB	Extended treatment duration
Lin et al. [54]	China	Prospective	2015–201	306	128	Patients with age ≥18 years, diagnosed TB	Age	Extended treatment duration
Wu et al. [55]	China	Retrospective	2007–2008	201	40	Pulmonary TB patients, residents	Age, gender, smoking history, pulmonary cavities, sputum smear status and TB treatment duration.	Extended treatment duration, recurrence
Mi et al. [56]	China	Retrospective	2011–2012	1589	189	Patients with diagnosed TB	Age, previous TB treatment	Extended treatment duration
Nandakumar et al. [57]	India	Retrospective	2010–2011	3116	667	Adult TB patients received DOTS therapy thrice-weekly	Age, gender, site and type of TB, smear status, HIV infection	Extended treatment duration
Mave et al. [58]	India	Prospective	2013–2019	574	225	Patients with age ≥18 years, confirmed pulmonary TB and DM	Age, gender, employment status, smoking, alcohol, BMI, smear status	Extended treatment duration, recurrence
Choi et al. [59]	Nigeria	Retrospective	2014–2016	1000	200	Diagnosed TB patients	Age, gender, HIV status, smoking	Extended treatment duration
Xhardo et al. [60]	Albania	Cross-sectional	2018–2019	140	13	Patients with diagnosed TB	Age, gender, BMI, smoking, alcohol, HIV status	Extended treatment duration
Baltas et al. [61]	UK	Retrospective	NR	838	126	Diagnosed TB patients	Age, gender, ethnicity, BMI, smoking, alcohol, comorbidities, previous TB treatment	Extended treatment duration
Tok et al. [62]	Malaysia	Retrospective	2014–2017	97,505	2464	Registered TB patients	Age, gender, education, residence, HIV, comorbidities	Extended treatment duration

ATT: anti-tuberculosis treatment; BMI; body mass index; CCI: Charlson Comorbidity Index; DOTS: Directly Observed Treatment-Short Course; HIV: human immunodeficiency virus; ICD: international classification of diseases; MDR-TB: multi-drug resistant tuberculosis; NR: not reported; PTB: pulmonary tuberculosis; WHO: World Health Organization; XDR-TB: extensively drug-resistant tuberculosis.

#### TB treatment outcomes

The TB treatment outcomes analysed in this study were categorized by WHO criteria. The outcomes analysed in this study were unsuccessful outcomes (extended treatment duration and recurrence). Since studies used different meanings for recurrence and relapse, we considered them as one-recurrence [64]. TB treatment outcomes were defined as extended treatment duration (TB patients with positive sputum culture results even after the fifth month of treatment or later or TB treatment failure patients with progression and worsening of infection in TB patients despite following the prescribed treatment protocol) and recurrence (TB symptoms reappear in TB patients after treatment, even if the patient was cured before) [64].

### Statistical analysis

Multivariable logistic regression results for TB unsuccessful treatment outcomes (extended treatment duration and recurrence) were preferably extracted. For pooling the estimates of DM impact on TB treatment outcomes, a fixed-effects model was used to calculate pooled hazards ratio (HR 95% CI). Heterogeneity was assessed between studies using *I*^2^ statistics. The studies reported higher heterogeneity if *I*^2^ values were greater than 50%. For TB treatment outcomes, the forest plots were also constructed. All analysis were conducted through licensed Statistical software package Stata V.16 (Stata Corp, College Station, TX).

## Results

### Search results

**Figure 1.**
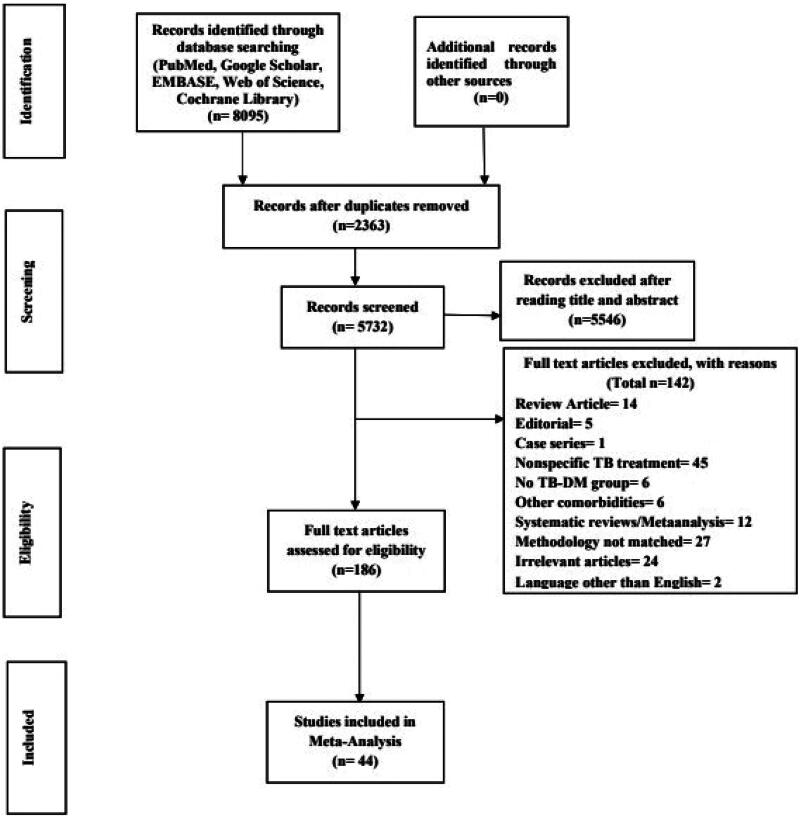
Study selection process in line with the PRISMA guidelines.

The databases searched a total of 8095 studies. After the removal of 2363 duplicates, 5732 articles were eligible for screening. After thoroughly screening the titles and abstracts, 186 studies were selected for full-text reading. The full text was not available for three studies, even after contacting the authors. A total of 44 studies were selected for inclusion in this meta-analysis. The search strategy is given in Figure 1.

### Study characteristics

Out of 49 studies included in this meta-analysis, nine studies were from India [24, 27, 30, 32, 36, 44, 47, 57,58], four from South Korea [1, 29, 31, 49], three from Taiwan [7, 23, 40], two from Ethiopia [2, 50], four from Mexico [28, 34, 42,43], six from China [17, 51,52, 54–56], one from Thailand [25], two from Malaysia [46, 62], two from Nigeria [35, 59], two from Brazil [37, 45], two from Georgia [38, 41] and one each from Indonesia [26], Canada [33], Pakistan [39], London [61], Armenia [48], Nepal [53] and Albania [60]. Among these, the study design of 23 studies was retrospective cohort, 19 studies were prospective, one study was a cross-sectional study and one study was a case-control study. The sample size for TB patients ranged from 90 to 199,571, and for TB-DM patients, the sample size varied from 13 to 47,952. The pooled sample size for TB patients in this meta-analysis was 623,989, and for TB-DM patients, it was 95,494.

### Impact of type 2 DM on TB treatment outcomes

#### Extended TB treatment duration

**Figure 2.**
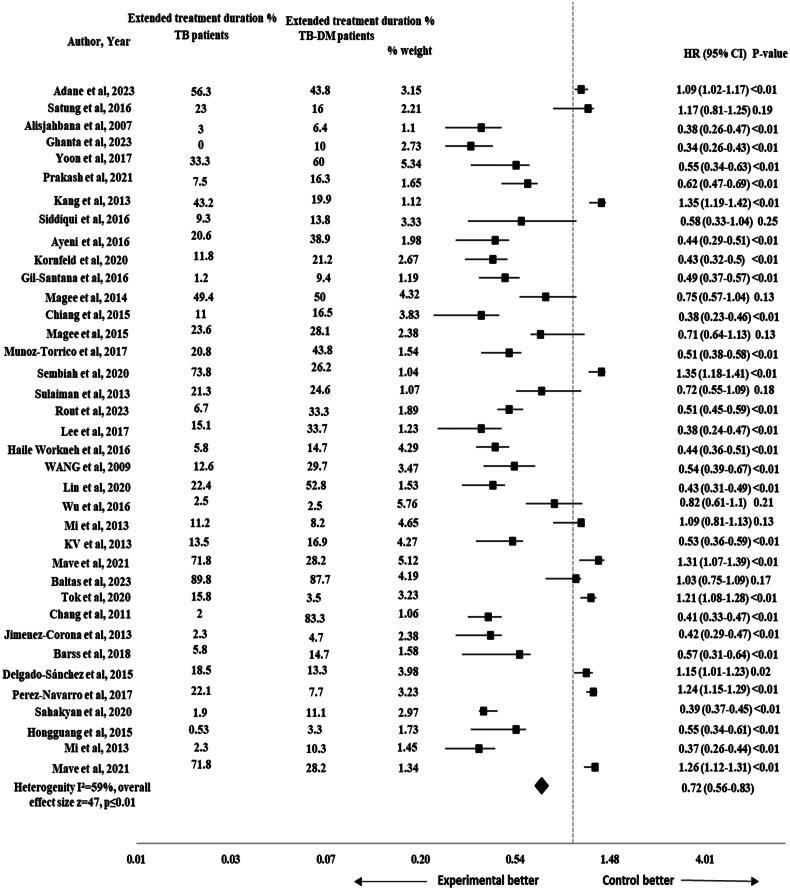
Forest plot for impact of DM on extended treatment duration in TB-DM comorbid patients.

**Figure 3.**
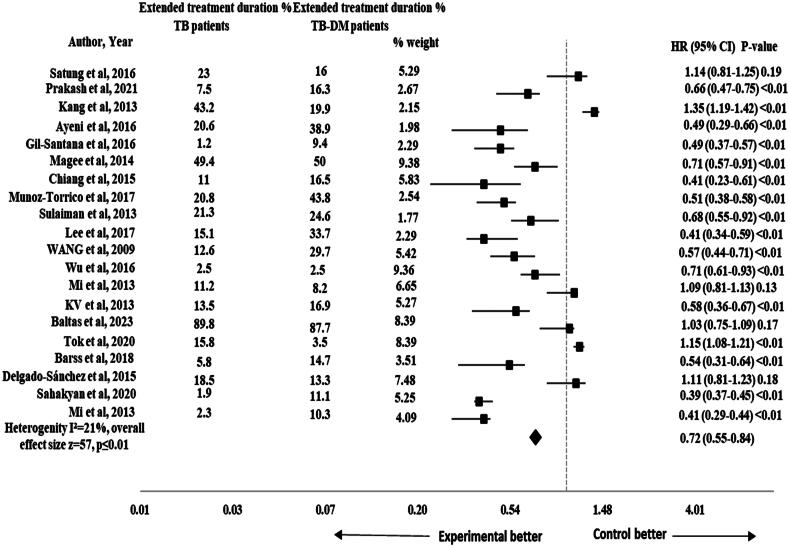
Forest plot of sub-group analysis for extended treatment duration in TB-DM comorbid patients.

The risk of extended treatment duration was reported in 37 studies [2, 7, 23, 25–38, 40–50, 52, 54–58, 60–62]. The pooled HR for the impact of DM on extended treatment duration was significant (HR 0.72, 95% CI 0.56-0.83), *p* ≤ .01 with 47 effect size and moderate heterogeneity (*I*^2^ = 59%) as shown in Figure 2. The subgroup analysis was performed by study design to assess the impact of different study designs on the pooled results. The results remained significant after performing sub-group analysis for extended treatment duration by study design (HR 0.72, 95% CI 0.55–0.84), *p* ≤ .01 and the heterogeneity was reduced to 21% (*I*^2^ = 21%) as shown in Figure 3.

#### Recurrence

**Figure 4.**
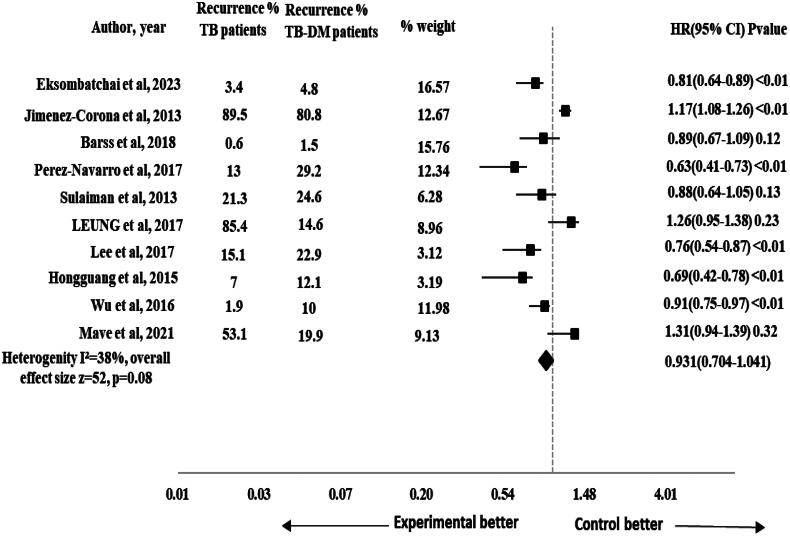
Forest plot for impact of DM on recurrence in TB-DM comorbid patients.

**Figure 5.**
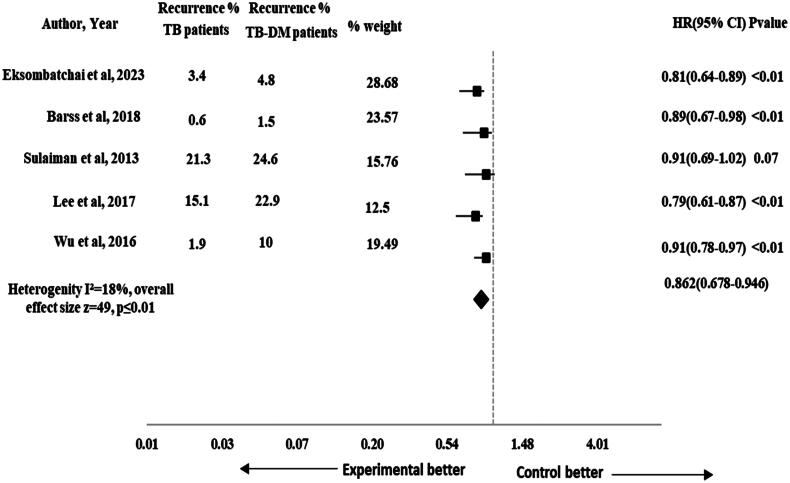
Forest plot of sub-group analysis for recurrence in TB-DM comorbid patients.

The risk of TB recurrence was reported in 10 studies [1, 17, 28, 33, 43, 46, 49, 52, 55, 58]. The pooled HR for the impact of DM on recurrence was non-significant (HR 0.931, 95% CI 0.704–1.041), *p* = .08 with 52 effect size. The heterogeneity observed across the studies was moderate (*I*^2^ = 38%) as shown in Figure 4. The subgroup analysis was performed by study design to assess the impact of different study designs on the pooled results. The results were significant for recurrence after performing sub-group analysis by study design (HR 0.862, 95% CI 0.678–0.946), *p* ≤ .01 and the heterogeneity was reduced to 18% (*I*^2^ = 18%) that showed consistent results across studies as shown in Figure 5.

### Composite TB treatment outcomes

**Figure 6.**
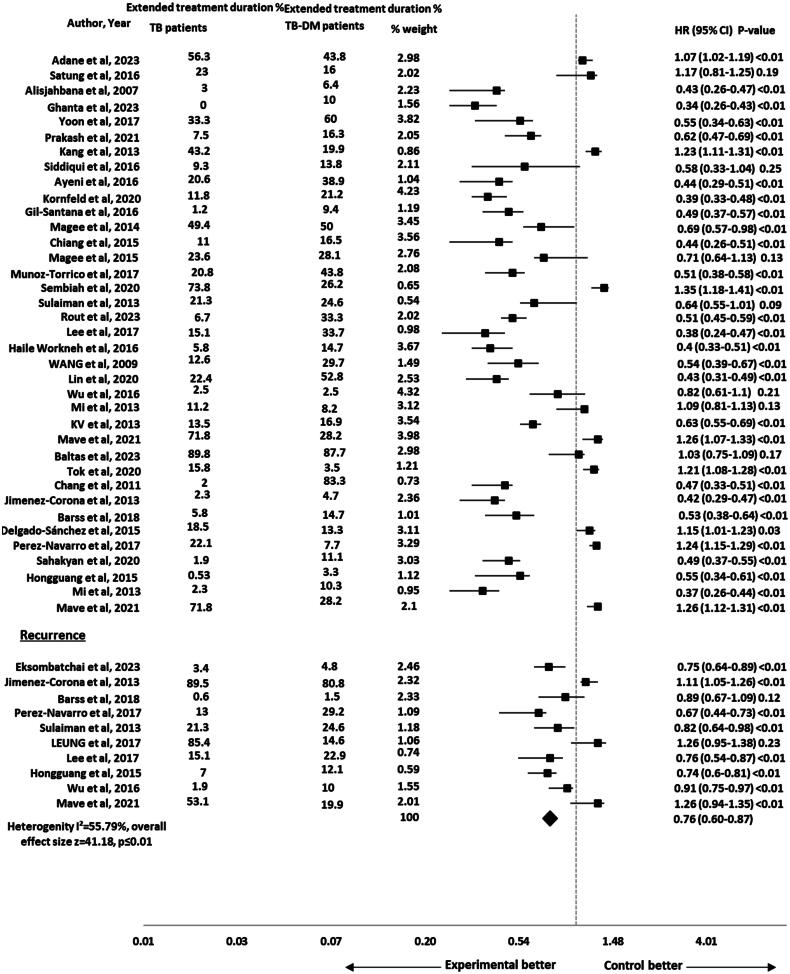
Forest plot for composite TB treatment outcomes in TB-DM comorbid patients.

The pooled HR (95% CI) for impact of DM on composite TB treatment outcomes (extended treatment duration and reoccurrence) was calculated as 0.76 (95% CI 0.60–0.87), *p* ≤ .01 with an effect size of 41.18. The heterogeneity observed among the included studies was moderate (*I*^2^ = 55.79%) as shown in Figure 6.

### Assessment of risk of bias

This meta-analysis used the NOS to evaluate the risk of bias in each individual study [63]. For studies analysing the impact of DM on TB treatment outcomes, the mean score of NOS was seven (out of a maximum of nine points), indicating the high quality of the studies included in this meta-analysis. The risk of bias in the included studies is provided in Table 2.

**Table 2.** Newcastle-Ottawa Scale.

Study	Study design	Selection	Comparability	Outcome/exposure	NOS score
Adane et al. [2], Ethiopia	Prospective	***	**	***	8
Chang et al. [23], Taiwan	Prospective	***		**	5
Eksombatchai et al. [1], South Korea	Retrospective	**	*	***	6
Viswanathan et al. [24], India	Retrospective	***	*	**	6
Satung et al. [25], Thailand	Retrospective	****	**	**	8
Alisjahbana et al. [26], Indonesia	Prospective	***	*	**	6
Ghanta et al. [27], India	Prospective	***	*	**	6
Jiménez-Corona et al. [28], Mexico	Prospective	****	**	**	8
Yoon et al. [29], South Korea	Prospective	****	**	*	7
Prakash [30], India	Retrospective	****	*	*	6
Kang et al. [31], South Korea	Retrospective	****	*	**	7
Siddiqui et al. [32], India	Prospective	***	**	*	6
Barss et al. [33], Canada	Retrospective	****	**	*	7
Delgado-Sánchez et al. [34], Mexico	Retrospective	****	**	*	7
Ayeni et al. [35], Nigeria	Retrospective	****	*	*	6
Kornfeld et al. [36], India	Prospective	***	**	**	7
Gil-Santana et al. [37], Brazil	Retrospective	****	**	*	7
Magee et al. [38], Georgia	Retrospective	****	**	*	7
Mukhtar and Butt [39], Pakistan	Prospective	****	**	**	8
Chiang et al. [40], Taiwan	Retrospective	****	**	***	9
Magee et al. [41], Georgia	Prospective	****	**	***	9
Muñoz-Torrico et al. [42], Mexico	Retrospective	****	*	***	8
Perez-Navarro et al. [43], Mexico	Prospective	****	**	***	9
Sembiah et al. [44], India	Prospective	****	*	***	8
Arriaga et al. [45], Brazil	Prospective	****	**	***	9
Sulaiman et al. [46], Malaysia	Retrospective	****	*	*	6
Rout et al. [47], India	Case control	***	*	*	5
Leung et al. [17], China	Prospective	****	**	***	9
Sahakyan et al. [48], Armenia	Retrospective	****	**	*	7
Lee et al. [49], South Korea	Retrospective	****	*	*	6
Haile Workneh et al. [50], Ethiopia	Prospective	****	**	**	8
You et al. [51], China	Retrospective	****	*	*	6
Hongguang et al. [52], China	Prospective	****	*	*	6
Wang et al. [7], Taiwan	Retrospective	****	**	*	7
Mahato et al. [53], Nepal	Prospective	****	**	**	8
Lin et al. [54], China	Prospective	***	**	*	6
Wu et al. [55], China	Retrospective	****	**	*	7
Mi et al. [56], China	Retrospective	****	**	**	8
Nandakumar et al. [57], India	Retrospective	****	**	***	9
Mave et al. [58], India	Prospective	****	**	**	8
Choi et al. [59], Nigeria	Retrospective	****	**	***	9
Xhardo et al. [60], Albania	Cross-sectional	****	**	*	7
Baltas et al. [61], London	Retrospective	****	**	***	9
Tok et al. [62], Malaysia	Retrospective	****	**	***	9

Each asterisk (*) represents a point that contributes to the overall quality score of the study. One * means one point.

## Discussion

This study conducted a meta-analysis to examine the impact of type 2 DM on TB treatment outcomes in pulmonary TB-DM comorbid patients. The analysis extensively reviewed articles specifically focusing on patients with TB treatment outcomes including extended treatment duration and recurrence who were given treatment following the DOTS therapy recommended by the WHO. Our findings explored that DM negatively influenced TB treatment outcomes. TB-non-DM patients had a lower risk of extended treatment duration and TB recurrence when compared with TB-DM comorbid patients.

This meta-analysis showed a significantly lower risk for extended treatment duration in TB-non-DM comorbid patients as compared to TB-DM patients (HR 0.72, 95% CI 0.56–0.83), *p* = .01 with moderate heterogeneity (*I*^2^ = 59%) across the studies. After performing sub-group analysis by study design, the risk for extended treatment duration remained lower in TB-non-DM comorbid patients as compared to TB-DM patients (HR 0.72, 95% CI 0.55–0.84), *p* = .01. The results were also found to be significant in previous study and systematic review [19, 21, 65]. But the results were inconsistent with previous studies that reported non-significant results [66–68]. The study’s small sample size could result in insufficient statistical power to detect minor differences. Statistical variability may also introduce uncertainty, contributing to non-significant results. Patient characteristics (age, gender, disease state) and healthcare system disparities could mask DM effects on treatment outcomes. Uncontrolled factors like socioeconomic status, healthcare access and adherence might complicate interpretation. These considerations highlight the diverse complexity of the results and suggest that the combined influence of these factors contributed to the non-significant relationship between DM and extended treatment duration in TB-DM comorbid patients [19].

Limitations in their study design or methodology might have affected their ability to detect a significant impact of DM on treatment prolongation. The study’s sample size and the characteristics of the patient population might not have been adjusted for detecting such an association. Similarly, another study might have had challenges related to patient enrolment, data collection or the duration of follow-up, potentially affecting their ability to identify a significant effect [38]. Additionally, the extended 5-year follow-up period in another study [61] reported confounding variables such as changes in treatment protocols, access to healthcare, or the presence of other comorbidities, which could make it challenging to clearly understand the association between DM and treatment prolongation. These factors highlight the need for accurate research design and interpretation when studying complex health outcomes, in order to obtain significant results.

Our study showed non-significant results for recurrence (HR 0.93, 95% CI 0.70–1.04), *p* = .08. The results were comparable with a previous study that reported no statistically significant impact of DM on recurrence in TB-DM comorbid patients [69]. On sub-group analysis by study design, the results were significant for recurrence (HR 0.86, 95% CI 0.67–0.94), *p* < .01. It showed that TB-non-DM patients were at lower risk of recurrence when compared with TB-DM comorbid patients. The previous systematic review and meta-analysis also reported a significant DM impact on recurrence in TB-DM comorbid patients [21, 70,71]. DM weakens the immune system of TB patients, making them more susceptible to TB infection. The pooled HR (95% CI) for impact of DM on composite TB treatment outcomes was 0.76 (95% CI 0.60–0.87), *p* = .01 in our study. Such disparities highlight the need for cautious interpretation. Due to the presence of type 2 DM, the cell-mediated immune functions are compromised in TB patients [72]. The type 2 DM if left uncontrolled can also impair the cytokine functions and disrupts type 1 cytokines responses [73]. The factors can contribute to unfavourable TB treatment outcomes including death, TB treatment prolongation and TB recurrence emphasizing the importance of future research for a more comprehensive understanding of the TB-DM comorbidity’s effect on TB treatment outcome including recurrence and TB treatment prolongation.

When comparing the results of this meta-analysis with previous research, it is noted that different studies have shown both significant and insignificant effects of DM on TB treatment results. These differences were discussed considering the limitations of the studies, like sample size, patient characteristics, methodological differences and uncontrolled factors. Still, despite these differences, the main findings of this study have given us a valuable understanding of how DM affects TB treatment outcomes, confirming the harmful impact of DM on different aspects of TB treatment. The strengths of this meta-analysis lie in its comprehensive analysis of a significant number of studies, focusing on a specific patient population treated under DOTS therapy. We focused on specific subtypes of DM and TB, providing a more refined understanding of their interaction. However, our study also had several limitations. We included relevant studies from different geographical regions by searching and reviewing the existing literature. There may be the possibility of publication bias, despite our efforts to include a comprehensive set of studies. The biasness may arise from underreporting of negative results or exclusion of studies with negative results leading to potential emphasis on significant findings only and such bias could affect the overall findings of our study. The method of diagnosis for type 2 DM was different in different studies. There was misclassification in the diagnosis of type 2 DM that can affect the results in examining the association between type 2 DM and TB. The glucose levels are increased temporarily during TB but some studies did not emphasize that either DM was diagnosed before TB or during TB or patients can be diagnosed as type 2 DM on the basis of short-term elevation of blood glucose levels. This factor can also impact our findings. The use of statistical methods for controlling diversity in the study designs can make the results unclear.

For future concern, this study highlights the importance of conducting more thorough research with large groups of people, using consistent methods, type 2 DM diagnosis and considering other variables that might affect the results. This would help gain a better understanding of how type 2 DM affects TB treatment outcomes.

## Conclusions

A negative impact of DM was found on recurrence and extended treatment duration in TB patients treated with DOTS therapy. Diabetes mellitus type 2 is responsible for the TB treatment prolongation and TB recurrence rates. By implementing effective management strategies and advancing research, the challenges can be mitigated arising due to the complex interaction between DM and TB.

## Data Availability

Data are available on request from the corresponding author.
